# FBG-Based Sensitivity Structure Based on Flexure Hinge and Its Application for Pipeline Pressure Detection

**DOI:** 10.3390/ma15165661

**Published:** 2022-08-17

**Authors:** Zhongyan Liu, Shunzhi Lu, Deguo Wang, Yanbao Guo, Lei Wu

**Affiliations:** 1 College of Mechanical and Transportation Engineering, China University of Petroleum (Beijing), Beijing 102249, China; 2 School of Petroleum Engineering, China University of Petroleum (Huadong), Qingdao 266580, China

**Keywords:** pipe pressure monitoring, non-intrusive structure, flexure hinge, mechanical lever, fiber bragg grating

## Abstract

With the widespread application of pipelines in engineering, more and more accidents occur because of pipeline leakage. Therefore, it is particularly important to continuously monitor the pipeline pressure. In this study, a non-intrusive and high-sensitivity structure based on FBG (Fiber Bragg grating) sensor is proposed. Firstly, the basic sensing theory of FBG and the state of a pipeline wall under inner pressure are analyzed. Then, structural sensitivity is deduced based on the flexure hinge and mechanical lever. Subsequently, finite element simulation for the whole sensitization structure is carried out, and optimal parameters are determined to obtain the maximum sensitivity. Finally, laboratory experiments are conducted to verify the function of the designed sensitivity structure. The experimental results show a good agreement with the simulation results. In the experiment, it can be found that the designed structure has a strain sensitivity of 9.59 *pm*/*με*, which is 11.51 times the pipeline surface strain. Besides, the structure is convenient to operate and has a good applied prospect for the engineering practice.

## 1. Introduction

It is well known that pipelines are widely used in transporting fluids due to the advantages of good stability and cost-effectiveness [1], which are especially suitable for oil and natural gas transportation. The transport medium is flammable, toxic, explosive, and corrosive; even a small leak will cause serious consequences [2,3]. Owing to the extension of service time of many pipeline infrastructures, especially in the case of long-distance transportation pipelines, it leads to more frequent leakage accidents. Therefore, the timely and accurate detection of pipeline leaks is crucial to avoid extreme safety accidents.

At present, there are two kinds of method for pipeline detection: the intrusive detection method and the non-intrusive detection method. For the intrusive detection method, the sensor is directly contact with the fluid, which will undermine the integrity and the service life of pipeline. Compared with the intrusive detection method, the non-intrusive sensor can be installed directly on the pipeline surface, and the pipeline will not be damaged. This means that the non-intrusive method has the advantages of preserving pipeline integrity and safely operations in a variety of applications. Hence, the non-intrusive method has become an important research direction.

In the field of engineering, there are multiple sensors widely used for pipeline pressure detection, such as resistance sensors [4,5], capacitive sensors [6,7], and FBG (Fiber Bragg grating) sensors, etc. Resistance sensors and capacitive sensors can monitor the pressure fluctuation from the change of resistance or capacitance values. The method has the advantages of high accuracy and strong stability. However, the sensor mentioned above is easily disturbed by the external environment and is generally fabricated into an intrusive structure that will damage the integrity of the pipeline. In comparison, FBG sensors have shown the advantages such as immunity to electromagnetic interference, resistance to corrosion, and small size [8,9,10,11,12]. Furthermore, FBG can establish a distributed measurement system, which can measure multiple signals simultaneously [13,14]. Because of the above advantages, FBG is widely used for structure monitoring in ocean engineering, oil, and gas transportation, with the measure of various parameters such as strain, pressure, and temperature. However, in practical engineering applications, the bare FBG is quite fragile and easily broken [15]. It requires adequate protection to be used for field application, especially in harsh industrial environments [16,17]. Besides, the strain sensitivity of directly pasted or embedded bare FBG sensors is about 1.21 *pm*/*µε* in theory [18]. If the variation of the pipeline pressure is small, the bare FBG cannot have sufficient sensitivity to realize a quick and accurate measurement of pipeline pressure change. Therefore, an FBG strain sensor with higher sensitivity and accuracy is urgently needed.

In recent years, in order to advance the sensitivity of bare FBG, some researchers have done a lot of work. Jia et al. [19] put a bare FBG directly bound on the surfaces of a pipeline for measuring circumferential strain; this measuring approach had no marked effect on the improvement of strain sensitivity. Li et al. [20] presented a strain sensor to enhance sensitivity by pasting the bare FBG on a substrate with a lever structure. The experimental results demonstrate that the developed FBG-based strain sensor achieves an enhanced strain sensitivity of 6.2 *pm*/*με*, and the strain sensitivity of the developed sensor is 5.2 times the strain sensitivity of a bare FBG strain sensor. The designed novel FBG-based strain sensor can be applied to micro-strain measurement in harsh industrial environments. Hu et al. [21] introduced a high-sensitivity FBG force sensor; two FBGs adhered to the surfaces of positive and negative strain bodies and the strain directions were opposite in the two bodies. The experimental results show that the sensitivity of the force sensor is about 4.8 times greater than an FBG directly adhered to the surface of the pipeline. Li et al. [22] developed an enhanced sensitivity FBG strain sensor with a sensitivity of 2.52 *pm*/*με*, and the strain measurement testing results were consistent with the theoretical calculation values. However, those structures mentioned above are cumbersome and inconvenient for engineering applications.

Additionally, some other researchers utilized substrates with flexure hinges to enlarge the sensitivity of the bare FBG. Liu et al. [23] designed a novel FBG strain sensor with high sensibility based on a flexure hinges bridge displacement magnification structure. The sensitivity was proved by simulation analyses and experimental results, and the results showed that the strain sensitivity of the structure was about 10.84 *pm*/*με*. Similarly, Peng et al. [24] also presented a novel sensitivity-enhanced structure based on lever mechanisms and flexure hinges. Calibration testing showed that the sensor has a strain sensitivity of 11.49 *pm*/*µε* within the measuring range of ±500 µε. In general, the above-mentioned structures can be used to accurately measure the strain of a mechanical structure surface. However, they are fragile and easy to be broken due to the weak flexure hinge and cannot be directly bound to the surfaces of the pipeline.

On the base of the above studies, some sensitivity FBG sensors were proposed. For example, Yan et al. [25] invented a non-intrusive strain-amplifying structure based on rhomboid structure; the bare FBG was attached to the surface of a long diagonal to monitor the strain. In the experiment, the strain can be amplified 5.6 times compared with the pipeline’s outside surface. However, this structure requires high ellipticity of the pipeline and is complicated to process and manufacture. Moreover, Shu et al. [26] designed an FBG sensor mounted on a flexure hinge substrate. The experimental results showed that the strain can be magnified by 4.75 times the strain on the pipeline surface. This design is easy to install. However, the substrate needs to be welded on the surface of the pipeline, which will destroy the anti-corrosion coating.

In view of the exposed problems, further improvements are necessary for the sensitization structure. Thus, this study presents a new sensitization structure based on flexure hinges and mechanical levers. Firstly, the principle of FBG is introduced. The mechanical model of a pipeline under internal pressure is built, and the stress-strain state is analyzed. Subsequently, the primitive model of the sensitization structure is designed, and the theoretical formula of strain magnification is deduced based on mechanical principles. Then, by the finite element method (FEM), we simulate the strain magnification of the designed structure. Finally, the laboratory experiment is conducted, and the experimental results are compared with the simulation results. The results demonstrate that the sensitization structure is feasible and reliable. Compared with the pipeline wall strain, the designed sensitization structure has a strain sensitivity of 9.59 *pm*/*με*, which is 11.51 times the pipeline surface strain.

## 2. Principle of FBG Sensors and Pipeline Mechanical Model

### 2.1. Basic Sensing Theory of FBG

FBG sensing technology takes optical fibers and light waves as the medium and carrier, respectively [27]. Optical fibers consist of a core layer, a cladding layer, and a coating layer. Because the refractive index of the cladding layer is smaller than the refractive index of the core layer, the light wave can travel along the core layer.

When the wide band light passes through, there is a selective transmission in which a narrow part of the spectrum is reflected and other wavelengths are transmitted. The reflected part of the spectrum is called the Bragg wavelength $\lambda_{B}$. According to the Maxwell’s equations and the coupled-mode theory of optical fibers, $\lambda_{B}$ can be expressed by the following formula [28,29]:

(1) $$\lambda_{B}=2n_{e}\cdot \Lambda$$

where $n_{e}$ is the effective refractive index and Λ is the period length of the optical fiber [30].

In engineering applications, the dependence of optical features of a grating structure on temperature and strain is used, which is reflected in the change in the Bragg wavelength $\lambda_{B}$. The relation between the Bragg wavelength change $\Delta \lambda_{B}$, the relative deformation ε, and the temperature variation ΔT is expressed by the following equation:

(2) $$\frac{\Delta \lambda_{B}}{\lambda_{B}}=\left(1-P_{e}\right)\cdot \varepsilon +\left(\alpha_{f}+\xi \right)\cdot \Delta T$$

where ξ is the coefficient of thermal expansion, $\alpha_{f}$ is the thermo-optic coefficient, and $P_{e}$ is the photo elastic coefficient.

### 2.2. Analyses of Pipeline Stress-Strain State

**Figure 1:**
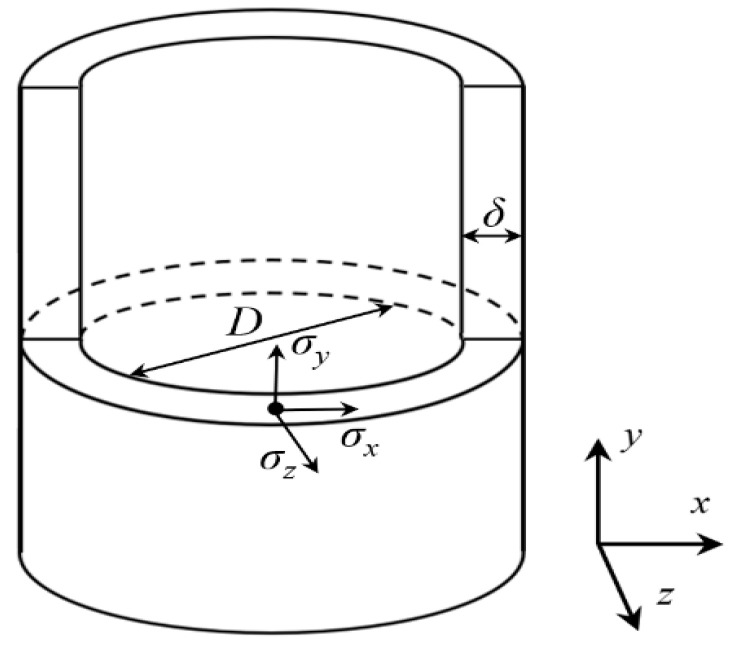
Stress state of thin-walled cylinder.

As shown in Figure 1, D is the inside diameter of the pipeline, and δ is the pipeline wall thickness. When D/δ>20, it can be defined as thin-walled circular tubes. Most of pipelines used in engineering can be classified as thin-walled circular tubes. Thus, applying an internal pressure *P* on the pipeline, principal stresses in three directions would be generated on the pipeline wall including $\sigma_{x}$, $\sigma_{y}$, and $\sigma_{z}$ ($\sigma_{x}$ is circumferential stress, $\sigma_{y}$ is axial stress, and $\sigma_{z}$ is radial stress). The principal stress at each point of the cylinder wall can be calculated according to the thin-walled cylinder theory as follows:

(3) $$\sigma_{1}{=\sigma }_{y}=\frac{PD}{4\delta }$$

(4) $$\sigma_{2}{=\sigma }_{x}=\frac{PD}{2\delta }$$

(5) $$\sigma_{3}{=\sigma }_{z}=0$$

Hence, based on the *Fourth theory of strength* [31], the equivalent stress $\sigma_{e}$ on the cylinder outside wall can be calculated as Equation (6):

(6) $$\sigma_{e}=\sqrt{\frac{1}{2}\left[{\left(\sigma_{1}-\sigma_{2}\right)}^{2}+{\left(\sigma_{2}-\sigma_{3}\right)}^{2}+{\left(\sigma_{3}-\sigma_{1}\right)}^{2}\right]}$$

According to the *Generalized Hooke Law*, the value of equivalent elastic strain $\varepsilon_{e}$ along the cylinder wall can be obtained as follows:

(7) $$\varepsilon_{e}=\frac{\sigma_{e}}{E}=\frac{\sqrt{\frac{1}{2}\left[{\left(\sigma_{1}-\sigma_{2}\right)}^{2}+{\left(\sigma_{2}-\sigma_{3}\right)}^{2}+{\left(\sigma_{3}-\sigma_{1}\right)}^{2}\right]}}{E}$$

where E is the elastic modulus of pipeline material (304 stainless steel).

## 3. Structural Design and Theoretical Calculation

### 3.1. Sensitization Structure Design

**Figure 2:**
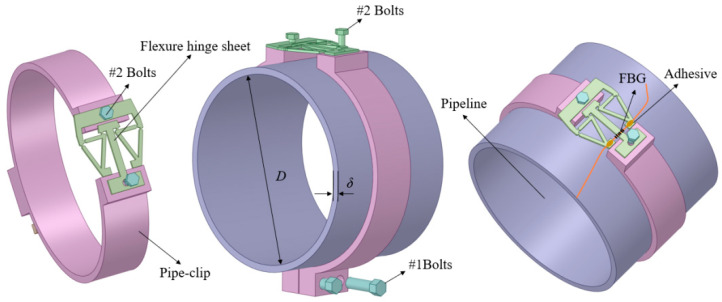
The proposed sensitization structure and assembly diagram.

The assembly diagram of the designed sensitization structure is shown in Figure 2. In detail, the sensitization structure is composed of a flexure hinge sheet, a pipe-clip, and bolts. Firstly, two-petal pipe-clips are fastened by #1 bolts, making the inner side of the pipe-clip closely attach to the pipeline wall to further make sure the pipeline wall strain is transmitting to the pipe-clip as much as possible. Then, the flexure hinge sheet is placed on the upper part of the pipe-clip, and both ends of the flexure hinge sheet are fixed to the pipe-clip by #2 bolts. The pipe-clip is set as a bridge to transmit strain from the pipeline wall to flexure hinge sheet. Then, an FBG is glued on the flexure hinge sheet by an adhesive with two ends fixed. More details are shown in Figure 2.

**Figure 3:**
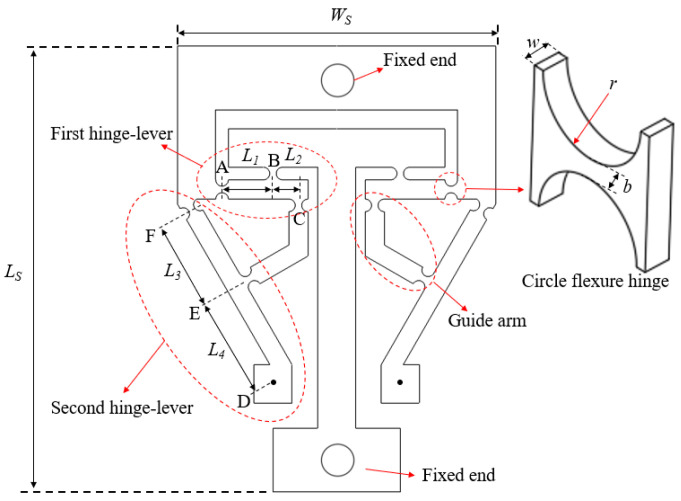
Size parameters of flexure hinge sheet.

As the core part of the designed structure, the flexure hinge sheet mainly consists of two levels of leverage (first hinge-lever and second hinge-lever), a circle hinge, a guide arm, and a fixed end, as shown in Figure 3. The distances between the points *A* and *B*, *B* and *C*, *E* and *F*, *D* and *E* are denoted by *L_1_*, *L_2_*, *L_3_*, and *L_4_*, respectively. Besides, *b* is the width of the narrowest part of the circle hinge. The radius and the thickness of the circle hinge are denoted by *r* and *w*, respectively. Moreover, the overall thickness of the flexure hinge sheet is *w*. The length and width of the whole flexure hinge sheet are *W_s_* and *L_s_*, respectively.

### 3.2. Static Analyses of Structures

**Figure 4:**
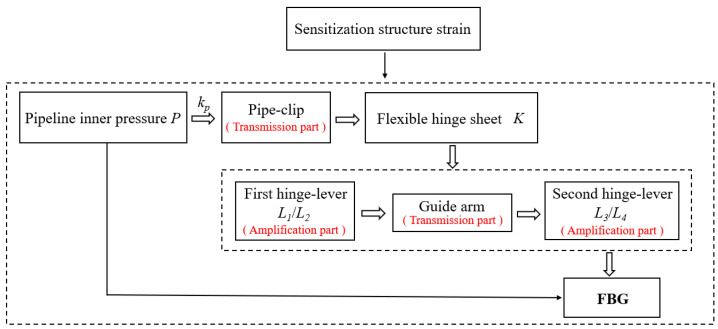
Flow diagrams of working principle of the sensitization structure.

For the proposed sensitization structure, the principle of strain transmission and amplification is derived in this section. The schematic diagram of working principle of the whole sensitization structure is shown in Figure 4. The relationship between the pipeline inner pressure *p* and the strain of FBG is obtained by calculating.

**Figure 5:**
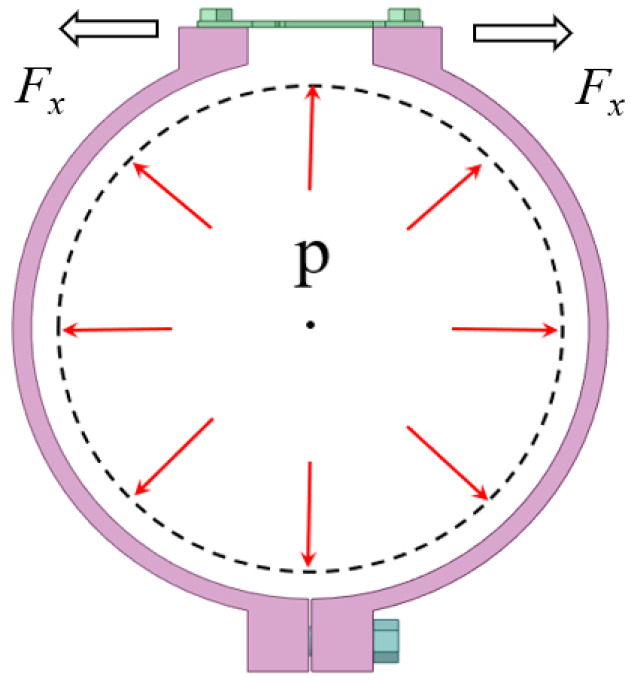
State of pipeline under internal pressure.

Firstly, as shown in Figure 5, the pipeline inner pressure *p* (Pa) causes the strain on the outer wall of pipeline, and then generate a tension *F_x_* (N) at the two ends of the pipe-clip. Because of the influence of friction and other factors, this can result in the strain’s incomplete pass to the pipe-clip. Hence, assuming there is a coupling coefficient *k_p_* between the pipeline inner pressure and the tension *F_x_* on the end of the pipe-clip, as shown in Equation (8). Then, the tension *F_x_* is transmitted to the ends of the flexure hinge sheet. The tension on the ends of the flexure hinge sheet is defined as *F_Ax_*. Clearly, *F_x_* and *F_Ax_* are a pair of interaction forces.

(8) $$F_{x}=k_{p}\cdot p$$

(9) $$F_{Ax}=F_{x}=k_{p}\cdot p$$

Subsequently, the beam *A–C* rotation about point *A* under the tension *F_Ax_* and generates a displacement at the point of *B* (∆*B*). *θ* is the deflection angle of *A*–*C*. Then, the displacement of point *D* (∆*D*) is amplified by the first hinge-lever, and the second hinge-lever. ∆*B* is along the *X*-axis direction. Since the guide arm has the function of guidance, ∆*D* is changed to the *Y*-axis direction by the guide arm. The amplification effect depends on the leverage ratios and the stiffness of the circle hinge.

**Figure 6:**
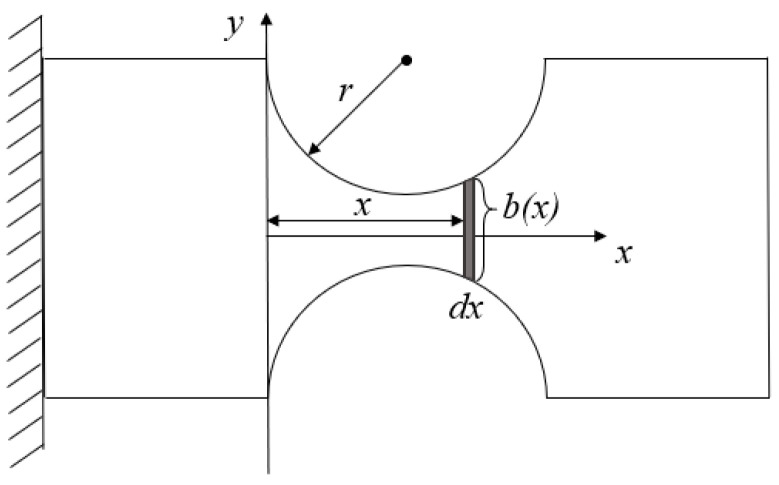
The micro-element of circle hinge.

Then, the relationship between the tension and displacement of the circle hinge is analyzed. As shown in Figure 6, a circle hinge is used as an example to calculate the actual displacement of the circle hinge. Simultaneously, for simplifying processes of the subsequent analysis. Assuming that:

(1) The displacement only occurs at the circle hinge, and the rest of the flexure hinge sheet can be considered to be rigid.
(2) The circle hinge only produces rotational displacement.

It shows that the width b(x) of the circle hinge varies with the change of *x*. Then, the width b(x) and the sectional area A(x) of the circle hinge can be calculated by using Equation (10).

(10) $$\{\begin{array}{c} b\left(x\right)=b+2r-2\sqrt{2rx-x^{2}}\\ A\left(x\right)=w\cdot b\left(x\right) \end{array}$$

The moment of inertia I(x) of the circle hinge is set up based on the theoretic of mechanics of materials as follows:

(11) $$I\left(x\right)=\frac{w\cdot b{\left(x\right)}^{3}}{12}=\frac{w\cdot {\left(b+2r-2\sqrt{2rx-x^{2}}\right)}^{3}}{12}$$

**Figure 7:**
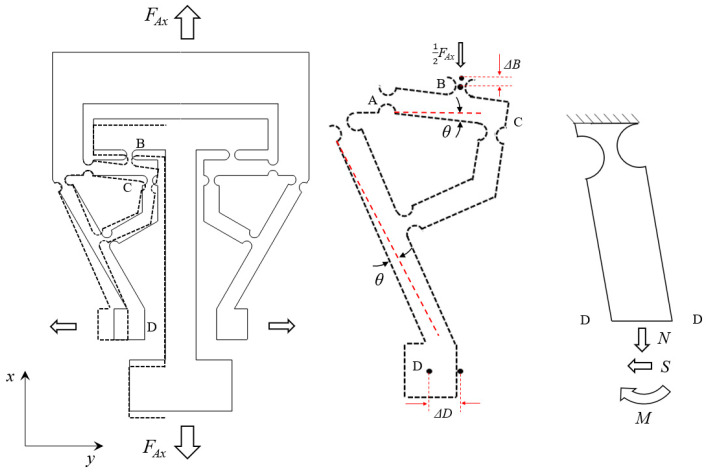
Mechanical diagram for one-half model.

Due to the flexure hinge sheet’s axially symmetric structure, it is reasonable to take half of the structure as a subject for analysis. As shown in Figure 7, there exists a bending moment M and a shear force S in the cross-section *D*. To simplify the calculation, coefficients *K_1_* and *K_2_* are introduced. The displacement of point *D* can be expressed as Equation (14).

(12) $$\{\begin{array}{c} U=U_{a}+U_{b}+U_{c}=\sum_{i=1}^{n}\left[\int_{0}^{l_{i}}\left(\frac{N_{i}^{2}}{2EA\left(x\right)}+\frac{S_{i}^{2}}{2GA\left(x\right)}+\frac{M_{i}^{2}}{2EI\left(x\right)}\right)dx\right]\\ S=K_{1}\cdot F_{Ax}=\frac{1}{2}K_{1}\cdot k_{p}\cdot p\\ M=K_{2}\cdot l_{1}\cdot F_{Ax}=\frac{1}{2}K_{2}\cdot k_{p}\cdot l_{1}\cdot l_{4}\cdot p \end{array}$$

where *E* is the elasticity modulus of the steel, and *G* is the shear modulus of the steel. $U_{a}$, $U_{b},$ and $U_{c}$ are the tensile, shear, and bending energy, respectively.

(13) $$\left\{\begin{array}{c} \frac{\partial U}{\partial M}=\frac{3r\int_{0}^{l}\left(\frac{{\left(K_{1}\cdot kp\cdot p\right)}^{2}\int_{-\pi /2}^{\pi /2}\frac{\cos\;\theta }{g{\left(\theta \right)}^{3}}d\theta }{G\cdot w\cdot (b+2r-2r\cos\;\theta )}+\frac{12{\left(K_{2}\cdot kp\cdot l_{1}\cdot p\right)}^{2}}{E\cdot w\cdot {\left(b+2r-2r\cos\;\theta \right)}^{3}}\right)dx}{Ewb^{3}\partial M}\\ \frac{\partial U}{\partial S}=\frac{3r\int_{0}^{l}\left(\frac{{\left(K_{1}\cdot k_{p}\cdot p\right)}^{2}\int_{-\pi /2}^{\pi /2}\frac{\cos\;\theta }{g{\left(\theta \right)}^{3}}d\theta }{G\cdot w\cdot (b+2r-2r\cos\;\theta )}+\frac{12{\left(K_{2}\cdot k_{p}\cdot l_{1}\cdot p\right)}^{2}}{E\cdot w\cdot {\left(b+2r-2r\cos\;\theta \right)}^{3}}\right)dx}{Ewb^{3}\partial S}\cdot \\ \Delta D=\frac{\partial U}{\partial M}+\frac{\partial U}{\partial S} \end{array}\right.$$

Finally, substituting Equation (13) into Equation (14), the strain on FBG can be derived.

(14) $$\begin{array}{cc} \varepsilon =\frac{\Delta D}{L}=\frac{3r}{Ewb^{3}L} & [\frac{\int_{0}^{l}\left(\frac{{\left(K_{1}\cdot k_{p}\cdot p\right)}^{2}\int_{-\pi /2}^{\pi /2}\frac{\cos\;\theta }{g{\left(\theta \right)}^{3}}d\theta }{G\cdot w\cdot (b+2r-2r\cos\;\theta )}+\frac{12{\left(K_{2}\cdot k_{p}\cdot l_{1}\cdot l_{4}\cdot p\right)}^{2}}{E\cdot w\cdot {\left(b+2r-2r\cos\;\theta \right)}^{3}}\right)dx}{\partial M}\\ & +\frac{\int_{0}^{l}\left(\frac{{\left(K_{1}\cdot k_{p}\cdot p\right)}^{2}\int_{-\pi /2}^{\pi /2}\frac{\cos\;\theta }{g{\left(\theta \right)}^{3}}d\theta }{G\cdot w\cdot (b+2r-2r\cos\;\theta )}+\frac{12{\left(K_{2}\cdot k_{p}\cdot l_{1}\cdot l_{4}\cdot p\right)}^{2}}{E\cdot w\cdot {\left(b+2r-2r\cos\;\theta \right)}^{3}}\right)dx}{\partial S}] \end{array}$$

where *L* is length of the FBG.

## 4. Simulation and Optimization

### 4.1. Analysis of Pipeline Wall Strain

**Figure 8:**
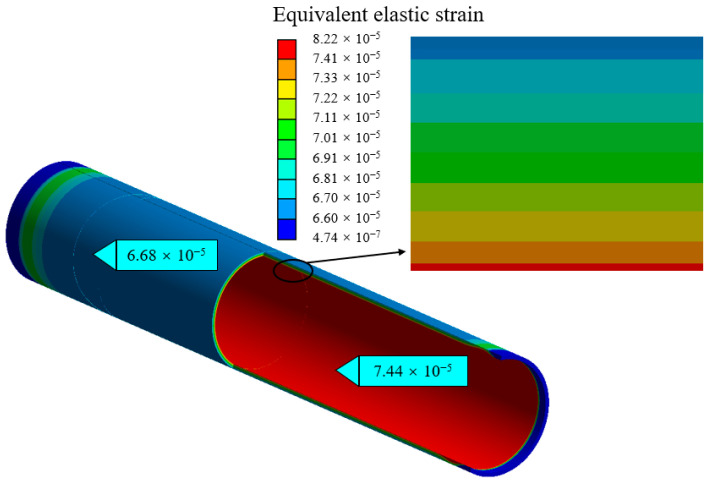
Distribution of equivalent elastic strain on pipeline wall.

Thin-walled pipeline is established as a model by *Ansys workbench*. The material of the pipeline is set as 304 stainless steel. The Young’s modulus and Poisson’s ratio of the stainless steel are set as 0.33 and 200 GPa, respectively. The length, outer diameter, and wall thickness are 500 mm, 89 mm, and 3 mm, respectively. A static pressure of 1.0 MPa is applied to the inside surface of pipeline, and both ends of the pipeline are set as fixed supports. Figure 8 shows the equivalent elastic strain distribution of the pipeline wall under a pressure of 1.0 MPa.

**Figure 9:**
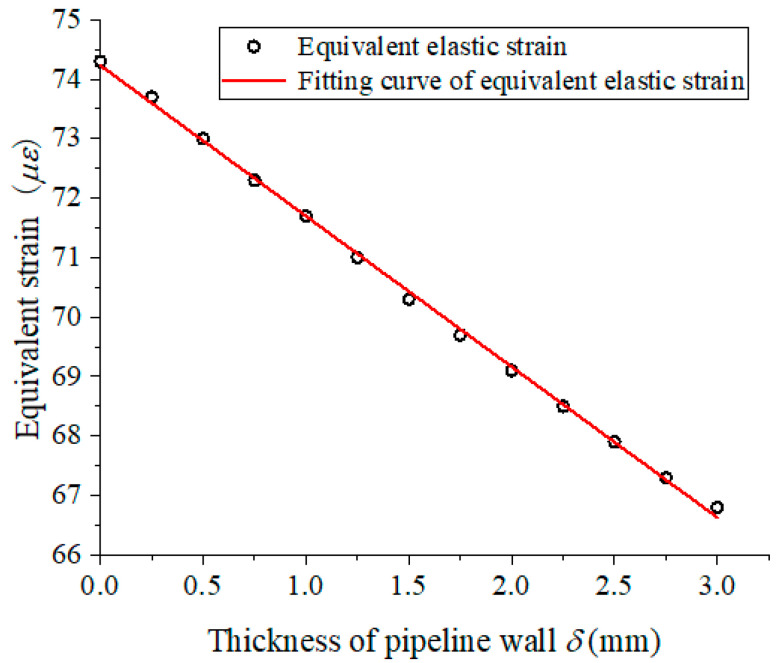
Strain along with the pipeline wall thickness variation.

As shown in Figure 8, the minimum value is 6.68 × 10^−5^ appearing on the pipeline’s outer wall, and the maximum value is 7.44 × 10^−5^ appearing on the pipeline’s inner wall. Figure 9 shows the strain curve along with the pipeline’s wall thickness variation. The equivalent elastic strain gradually decreases along with the wall thickness increasing, and the equation of pipeline wall strain and wall thickness is obtained by using linear fitting, as shown in Equation (15). The fitting coefficients *R*^2^ is 0.9988 and shows the wall thickness and strain coincide with a linear relation. The theoretical strain value calculated according to the formula Equation (7) is 6.12 × 10^−5^; the outer wall strain obtained by finite element is 6.68 × 10^−5^. The relative errors of both values are below 8%; this indicates that the result of the simulations consists well with the theoretical strain value.

(15) $$\varepsilon_{e}=-2.5340\times 10^{-6}\times \delta_{d}+74.231\times 10^{-6}\;(R^{2}=0.9988)$$

### 4.2. Sensitivity Analysis of the Critical Dimensions

**Figure 10:**
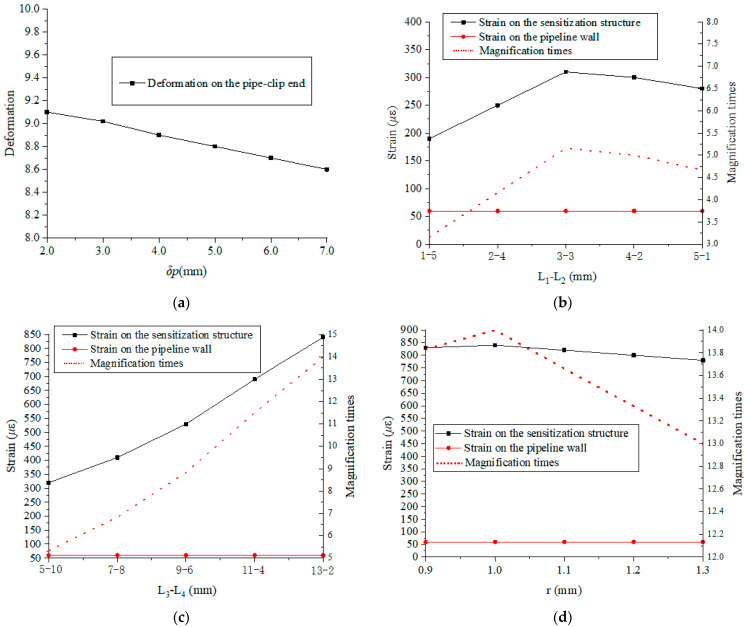
Sensitization changes with different parameters. (**a**) *δ_p_*, (**b**) *L_1_-L_2_*, (**c**) *L_3_-L_4_*, (**d**) *r*.

The critical dimensions (pipe-clip thickness *δ_p_*, first hinge-lever *L_1_-L_2_*, second hinge-lever *L_3_-L_4_*, and circle hinge radius *r*) are simulated to further analyze the sensitization ability of the proposed structure. Firstly, the sensitization structure is modeled according to the initial values provided in Table 1. The whole structure is assembled according to Figure 2. Then, a pressure of 1.0 MPa is applied to the inner surface of pipeline, and the correlation between the pipeline’s outer wall strain and the sensitization structure strain is observed and analyzed. The magnification times are defined as the ratio of strain on the sensitization structure and pipeline wall. The strain value on the pipeline wall, sensitization structure, and magnification times are presented in Figure 10.

For the pipe-clip, it is more intuitive to observe amplification effect by deformation. As shown in Figure 10a, the deformation sensitivity is affected by the pipe-clip thickness *δ_p_*. As the pipe-clip thickness increases, the deformation value gradually decreases, and the deformation on the pipe-clip end is approximately linear with the pipe-clip thickness *δ_p_*, it means that the value *δ_p_* should be as small as possible. However, a small value of *δ_p_* will increase the processing difficulty. Hence, the value of *δ_p_* is set to be 3.0 mm, which is consistent with pipeline wall thickness.

The effect of the first hinge-lever *L_1_-L_2_* is shown in Figure 10b; the strain on the sensitization structure increases from 190 *με* to 310 *με* with the increment of *L_1_* when *L_1_* < 3 mm, and then decreases to 280 *με* when *L_1_* > 3 mm. As we know, the strain on the pipeline wall is stable. Since the strain on the pipeline wall is much less than the strain on the sensitization structure, the magnification times shows an almost similar tendency to the variation of strain on the sensitization structure. Finally, the value for *L_1_-L_2_* is determined to be 3 mm-3 mm.

For the second hinge-lever *L_3_-L_4_*, the strain on the sensitization structure increases from 320 *με* to 840 *με* with the increment of *L_3_* (the decrease of *L_4_*), as shown in Figure 10c. The strain on the pipeline wall is stable at a certain value of 60 *με*. The magnification times also increase with the increment of *L_3_,* and the magnification times increase to 14 when *l_3_* = 13 (*L_4_* = 2). Hence, the value of *L_3_-L_4_* is set as 13 mm-2 mm.

The sensitivity with the variation of *r* is plotted in Figure 10d. The strain on the sensitization structure increases from 830 *με* to 840 *με*, with the radius *r* increasing to 1.0 mm. Then, the strain on FBG gradually decreased to 780 *με* when *r* > 1.0 mm and the strain on the pipeline wall remained static. This indicates that the sensitivity of proposed structure is very significant when *r* = 1.0 mm, and the magnification times increase to 14.0 when *r* = 1.0 mm.

Based on the above optimization results, the dimensions are determined as follows: *δ_p_* = 3 mm, *L_1_-L_2_* = 3-3 mm, *L_3_-L_4_* = 13-2 mm, *r* = 1 mm. Moreover, considering the difficulty of processing and assembly, the remaining parameters are determined as shown in Table 2. Finally, the dimension parameters and material properties of the proposed sensitization structure are confirmed.

### 4.3. Sensitization Ability of the Structure

**Figure 11:**
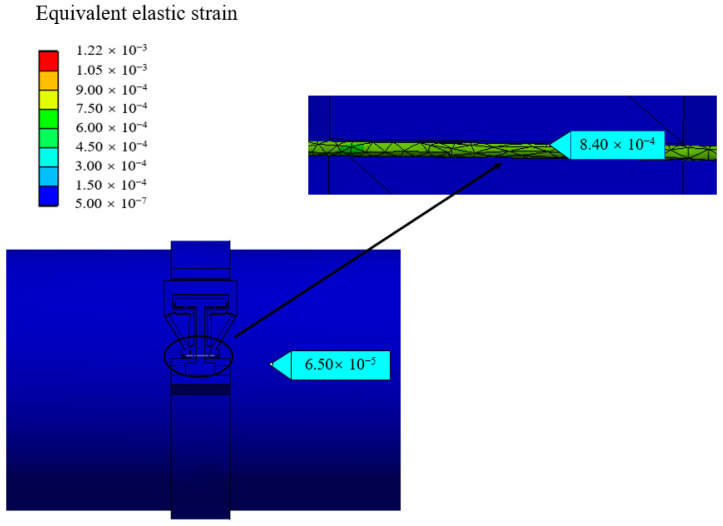
Strain distribution of pipeline wall and sensitization structure.

According to the dimensions provided in Table 2, the optimized structure is established. Then, a pressure of 1.0 MPa is applied on the inner surface of pipeline, and both ends of the pipeline are set as fixed supports. The friction conditions between the pipeline and pipe-clip are set as frictional. Both ends of the flexure hinge sheet are fixed to the pipe-clip by bolt connections. The equivalent elastic strain values on pipeline wall and sensitization structure are obtained and shown in Figure 11. The sensitization structure exhibits a uniform strain distribution with a value of 8.40 × 10^−4^. Meanwhile, the strain on the pipeline wall is 6.50 × 10^−5^; the difference in strain values is obvious.

**Figure 12:**
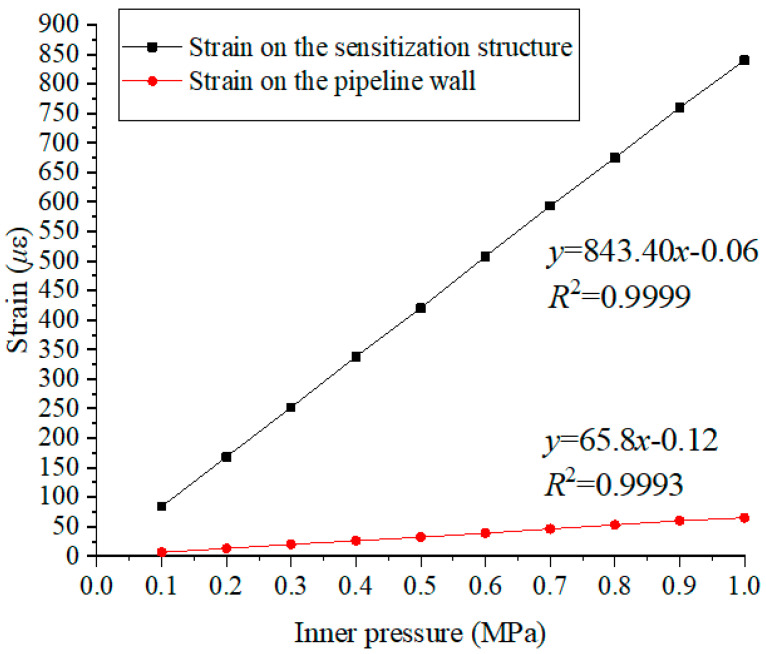
Strain distribution under different pressure values.

In order to get a more precise results of magnification times of the sensitization structure, the internal pressure is increased from 0.1 MPa to 1.0 MPa with 0.1 MPa interval. The strain values under different pressure are shown in Figure 12, and the curve between the pressure values and strain results is plotted. For sensitization structure, the slope of the fitting curve is 843.40, while for pipeline wall strain, the slope of the fitting curve is 65.80. The fitting coefficient *R*^2^ are 0.9999 and 0.9993, respectively. Hence, it can be found that there is a stable and good linear relationship between the pressure and the strain. Furthermore, the magnification times of this sensitization structure is 12.81 (843.40/65.80 = 12.81).

## 5. Experimental Testing and Analyses of Results

### 5.1. Experimental System Setup and Results

**Figure 13:**
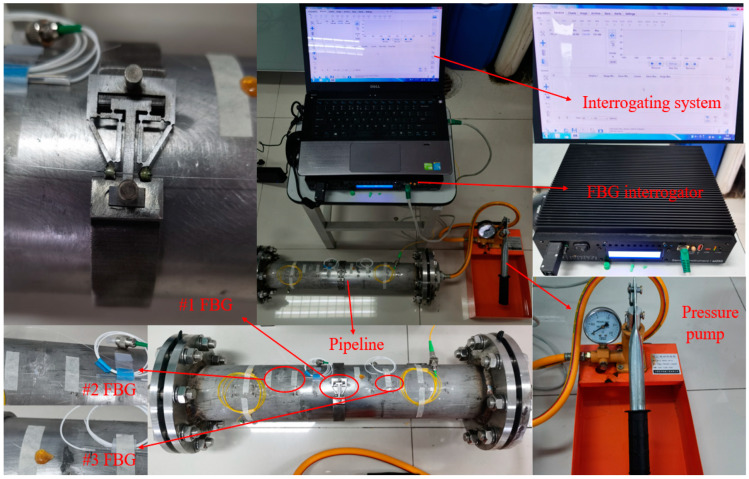
Experimental system set up.

The experimental system for pipeline internal pressure detection is built up as shown in Figure 13, which includes a stainless-steel pipeline (with a length of 500.0 mm, a wall thickness of 3.0 mm, and an outer diameter of 89.0 mm), a sensitization structure, a pressure pump with pressure gauge (with a maximum pressure of 3.0 MPa), an FBG demodulator (the model is si255, produced by the TONGWEI Technology Co., Ltd., Shenzhen, China), a spectrum software, and several FBGs. The detailed parameters of FBG are listed in Table 3. Both the ends of pipeline are sealed by flanges, and the sensitization structure is mounted in the middle of the pipeline. Then, by using 353 ND glue, two FBG sensors are attached to the surface of sensitization structure and pipeline outer wall, respectively.

The FBG on the sensitization structure is marked as #1FBG, which is parallel to the pipeline axis. The FBG on pipeline wall is marked as #2FBG and is perpendicular to the pipeline axis. The laboratory temperature is maintained at about 25.0 degrees. In order to reduce the effect of the temperature, #3FBG, which is being used for temperature compensation, only has one fixed end. The arrangement of #1FBG, #2FBG, and #3FBG is shown in Figure 13. Then, pressurizing the pipeline pressure from 0 MPa to 1.0 MPa with a pressure pump, the center wavelength variations of #1FBG and #2FBG with 0.1 MPa intervals are recorded. To verify the accuracy and reliability of the proposed structure, two samples are tested, and two circle processes of increasing pressure and decreasing pressure are conducted for every sample. Then, the average values of each pressurized cycle or depressurized cycle are recorded and shown in Table 4.

### 5.2. Analyses of Experimental Results

**Figure 14:**
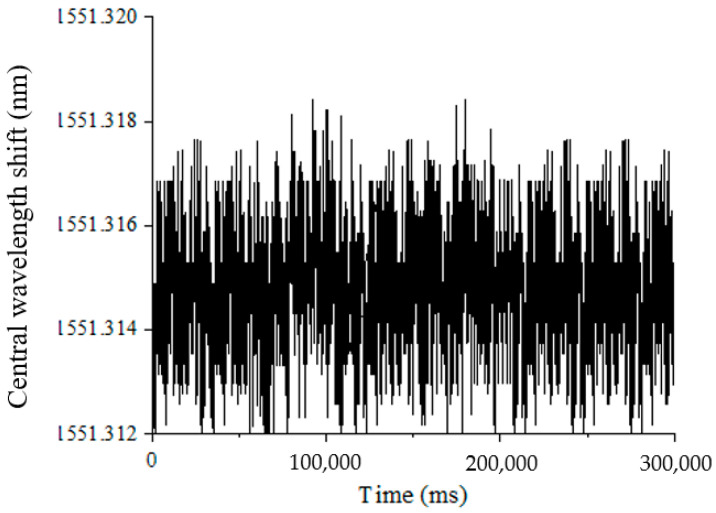
Central wavelength variation of #3FBG caused by temperature.

To verify the temperature effect on the testing results, the central wavelength variation of #3FBG in five minutes is recorded, as shown in Figure 14. Obviously, the central wavelength of the #3FBG is almost constant, which means that the temperature remains stable (Δ*T* = 0). Thus, it is reasonable to ignore the temperature effect when testing the function of the proposed structure.

**Figure 15:**
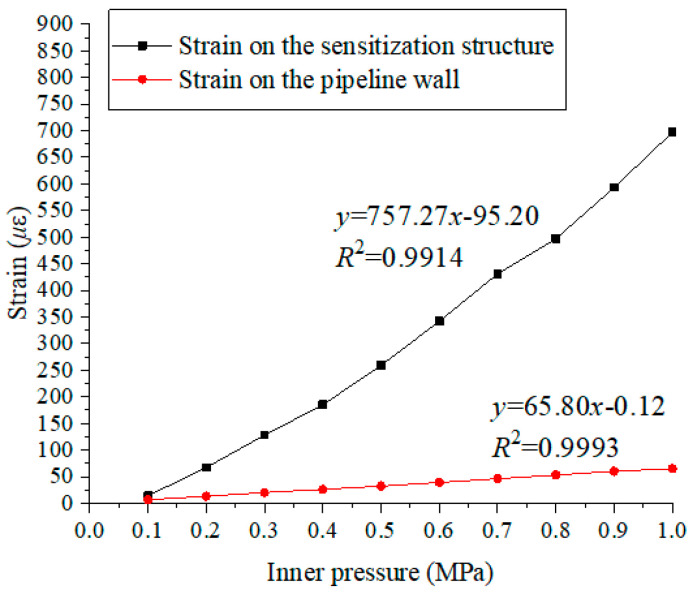
Central wavelength variation of #1FBG and #2FBG.

According to the center wavelength variations obtained by #1FBG and #2FBG, the strain values on the proposed sensitization structure and pipeline wall can be obtained and shown in Figure 15. As shown in Figure 15, it can be seen that the center wavelength shift of #1FBG (black line) shows a linear relationship with the change of the pipeline’s inner pressure as the pressure increasing, the center wavelength gradually increases. By using linear fitting, the slope of the black line is about 757.27. This means that the strain on the sensitization structure can increase to 757.27 *με*/MPa (757.27/1.20 = 631.06 *pm*/MPa) when the pressure increases by 1.0 MPa. From #2FBG (red line), it can be seen that there also exists a significant linear correlation between the pipeline’s wall strain and inner pressure. The slope of the red line is about 65.80 *με*/MPa. The related fitting coefficients *R*^2^ are 0.9914 and 0.9993, respectively. It can be concluded that the strain value is obviously expanded by the sensitization structure compared with the wall strain. The sensitivity of the proposed sensitization structure is approximately 9.59 *pm*/*με*, and the strain on the proposed sensitization structure is 11.51 (757.27/65.80 = 11.51) times the strain on the pipeline’s outside surface.

This shows that the structure has a high sensitivity for pipeline pressure monitoring. Meanwhile, FBG can establish a distributed measurement system, and multiple sensors can be connected in series or parallel. Therefore, multiple FBG sensors can be arranged along the pipeline to monitor the pressure in different positions.

## 6. Conclusions

This study presented a strain amplifying structure based on FBG. The structure with combinations of flexure hinge and mechanical lever, mainly includes two parts: a flexure hinge sheet and a pipe-clip. Firstly, the principle of FBG and stress-state of the pipeline are introduced. Then, a novel structure is designed, and the strain amplification effect is conducted by mechanical analyses. Subsequently, the parameter optimization for the designed structure is operated by FEM, and the optimal size is obtained. Finally, the sensitivity of the structure is confirmed through experiments and the results are analyzed, and the experimental results are consistent with the simulation results. The results demonstrate that this structure can obviously magnify the strain on the pipeline’s walls. It has a strain sensitivity of 9.59 *pm*/*με*, which is 11.51 times than the pipeline surface strain. In short, the proposed structure can achieve non-intrusive detection and has a good application prospect in structural health monitoring. Moreover, it has a small size and high sensitivity and has very high practical value.

## Figures and Tables

**Table 1:** Initial dimensions of sensitization structure.

Parameter	*δ_p_*	*L_1_-L_2_*	*L_3_-L_4_*	*r*	*w*	*W_s_*	*L_s_*
Initial dimension	3 mm	3–3 mm	6–7 mm	1 mm	1 mm	20 mm	30 mm

**Table 2:** Dimensions and material parameters of the sensitization structure.

Symbol	Description (Unit)	Value
*D*	Inside diameter of pipeline (mm)	83.0
*L_1_-L_2_*	First hinge-lever (mm)	3.0–3.0
*L_3_-L_4_*	Second hinge-lever (mm)	13.0–2.0
*δ*	Wall thickness of pipeline (mm)	3.0
*w*	Thickness of flexure hinge sheet (mm)	1.0
*δ_p_*	Thickness of pipe-clip (mm)	3.0
*L_s_*	Length of the flexure hinge sheet (mm)	30.0
*W_s_*	Width of the flexure hinge sheet (mm)	20.0
*α_f_*	Thermal expansion coefficient of fiber (°C)	0.5 × 10^−6^
*ξ*	Thermo-optical coefficient of fiber (°C)	6.4 × 10^−6^
*E*	Young’s modulus of the sensitization structure (GPa)	200.0
*μ*	Poisson’s ratio of the sensitization structure	0.33

**Table 3:** Detailed parameters of FBG.

	Central Wavelength [nm]	FBG Length [mm]	Reflectivity [%]
#1FBG	1551.705	10	91.09
#2FBG	1551.500	10	93.69
#3FBG	1551.315	10	95.24

**Table 4:** Experimental data and relative errors.

Pressure (MPa)			0.1	0.2	0.3	0.4	0.5	0.6	0.7	0.8	0.9	1.0
Pressurized cycle (*µε*)		Sample 1	11.67	55.83	106.67	154.17	215.83	285.00	359.17	414.17	494.17	580.83
		Sample 2	10.83	54.17	104.17	155.00	214.17	284.17	358.33	417.50	490.83	577.50
	Relative error of the slope/%		0.32									
Depressurized cycle (*µε*)		Sample 1	12.50	57.50	108.33	158.33	212.50	282.50	364.17	408.33	487.50	580.00
		Sample 2	12.50	57.50	105.83	152.50	210.83	282.50	360.83	422.50	487.50	575.00
	Relative error of the slope/%		0.43									

## Data Availability

The data that support the findings of this study are available from the corresponding author upon reasonable request.
